# Oxidized Low-Density Lipoprotein Induces Apoptosis in Cultured Neonatal Rat Cardiomyocytes by Modulating the TLR4/NF-κB Pathway

**DOI:** 10.1038/srep27866

**Published:** 2016-06-09

**Authors:** Xiantao Wang, Yuhan Sun, Huafeng Yang, Yuanxi Lu, Lang Li

**Affiliations:** 1 Department of Cardiology, the First Affiliated Hospital of Guangxi Medical University, Nanning 530021, China

## Abstract

This study was designed to investigate the apoptosis induced by oxidized low-density lipoprotein (ox-LDL) in cultured neonatal rat cardiomyocytes and explore the possible mechanisms. We evaluated whether ox-LDL-induced apoptosis depended in part on the activation of toll-like receptor-4(TLR4)/Nuclear factor κB (NF-κB) signaling pathway. Cells were cultivated with and without ox-LDL. Cell apoptosis was evaluated by flow cytometry. Immunofluorescence, western blot analysis and quantitative real-time polymerase chain reaction (qRT-PCR) were conducted to assess protein or mRNA expressions. Resatorvid (TAK-242), an exogenous synthetic antagonist for TLR4, was used to inhibit TLR4 signal transduction. Dose- and time-dependent apoptotic index of cardiomyocytes occurred after ox-LDL treatment. Incubation of cardiomyocytes with ox-LDL (50 μg/mL) for 24 hours increased TLR4 and NF-κB expressions significantly. Decrease of Bcl-2/Bax protein ratio, activation of caspase-3 and 9 were also detected. Ox-LDL-induced cardiomyocyte apoptosis, TLR4 and NF-κB expressions were attenuated by pretreatment with TAK-242. In conclusion, our findings indicate that the apoptosis induced by ox-LDL in cultured neonatal rat cardiomyocytes at least in part by modulating the TLR4/NF-κB signaling pathway.

Atherosclerosis is now recognized as a chronic inflammatory disease, and the main character is a focal atheromatous plaque formation^1^,^2^. Oxidized low-density lipoprotein (Ox-LDL) is oxidatively modified form of LDL and it contributes to the atherosclerotic plaque formation and progression. Ox-LDL is toxic to cells via trigger of oxidative mechanisms and it is quite clear that few cells are completely refractory to the toxicity of ox-LDL^3^,^4^. The plasma level of ox-LDL was discovered to be a prognostic predictor of mortality in patients with chronic congestive heart failure, and a significant negative correlation existed between ox-LDL level and left ventricular ejection fraction^5^. Ox-LDL was found to induce severe cell damage in ventricular myocytes including apoptosis and irregular electrical activity^6^,^7^. The toll-like receptor-4 (TLR4) plays a crucial role in the cell response to ox-LDL exposure and nuclear factor κB (NF-κB) activation is an early step downstream of TLR4^8^,^9^,^10^. Apoptosis is an essential feature of various diseases including atherosclerosis. However, to our knowledge, there have been no reports about the role of TLR4 in the apoptosis induced by ox-LDL in cultured neonatal rat cardiomyocytes.

In the present study, we investigated the effect of ox-LDL on cardiomyocyte apoptosis. We found that ox-LDL induced cardiomyocytes apoptosis via TLR4/NF-κB signaling pathway and inhibition of TLR4 signal transduction by resatorvid (TAK-242) then significantly suppressed ox-LDL-induced cardiomyocyte apoptosis. Our results reveal the signaling transduction of ox-LDL-induced cardiomyocyte apoptosis, and inhibition of TLR4 signaling pathway may provide new therapeutic options for myocardial injury in atherosclerotic cardiovascular diseases.

## Results

### Dose- and time-dependent apoptosis in cardiomyocytes by ox-LDL

**Figure 1:**
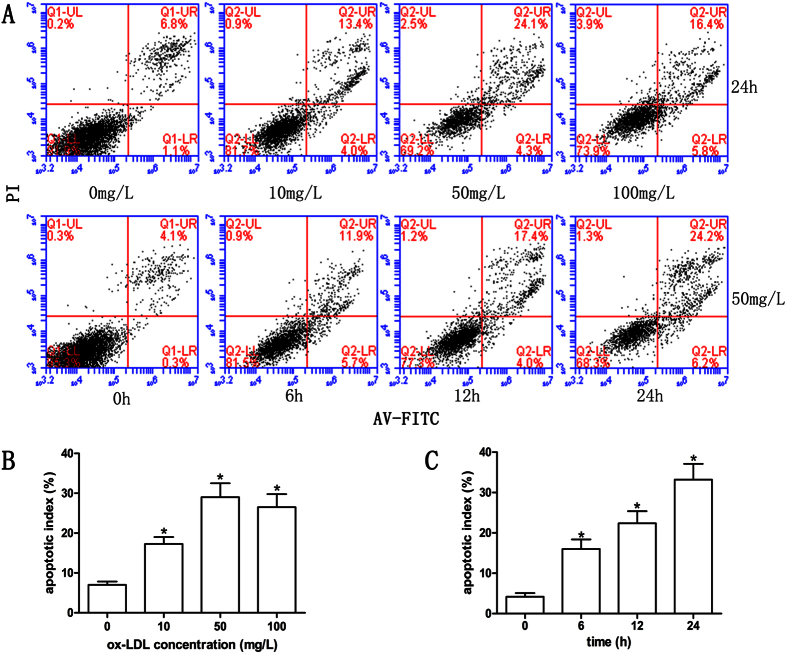
Dose- and time-dependent manner of apoptosis in cardiomyocytes by ox-LDL. (**A**) Cell apoptosis was assessed by flow cytometry using Annexin V-FITC Apoptosis Detection Kit with PI (the upper left quadrant represents necrotic cells; the upper right quadrant contains the later apoptotic cells; the lower left quadrant shows viable cells; the lower right quadrant denotes the early apoptotic cells). (**B,C**) Cells were incubated with different concentrations of ox-LDL (0, 10, 50, 100 mg/L) for 24 h or with 50 mg/L ox-LDL for 0, 6, 12 and 24 h respectively. Data are mean ± standard deviation (n = 4 for each group). *P < 0.05 versus the control group.

To confirm the apoptosis-inducing effect of ox-LDL, the cultured cardiomyocytes were incubated with different concentrations of ox-LDL (0, 10, 50, 100 mg/L) for 24 h or with 50 mg/L ox-LDL for 0, 6, 12 and 24 h respectively at identical environment, and cell apoptosis study was conducted by flow cytometry assay. The results showed that ox-LDL caused cardiomyocytes apoptosis in a dose- and time-dependent manner (Fig. 1), which provide information about the suitable concentration of ox-LDL for further studies.

### TLR4 is involved in ox-LDL induced apoptosis in cardiomyocytes

**Figure 2:**
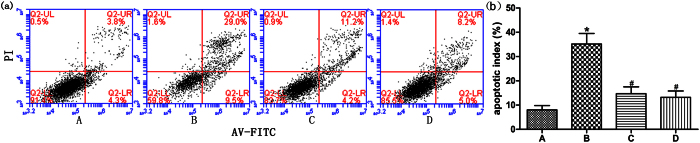
Apoptotic index of cardiomyocytes following inhibition of the TLR4/NF-κB signaling pathway detected by flow cytometry. (**a**) Cell apoptosis was assessed by flow cytometry. (**b**) Changes of apoptotic index in each group. (A) Control group, (B) OX-LDL group, (C) OX-LDL + TAK-242 group, (D) TAK-242 group. Data are expressed as mean ± standard deviation(n = 4). *P < 0.05 versus the control group; ^#^*P* < 0.05 versus ox-LDL-treated group. TLR4, toll-like receptor 4.

To investigate whether TLR4 is involved in the positive results of apoptosis in cultured cardiomyocytes after ox-LDL stimulation, we pretreated cells with TAK-242 for 1h, an exogenous synthetic antagonist for TLR4, followed by incubation with ox-LDL (50 mg/L for 24 h). As shown in Fig. 2, TAK-242 significantly inhibited ox-LDL-induced apoptosis in cultured cardiomyocytes. Thus, TLR4 might play an important role in ox-LDL induced apoptosis in cardiomyocytes.

### Expression of TLR4 in cardiomyocytes by ox-LDL

**Figure 3:**
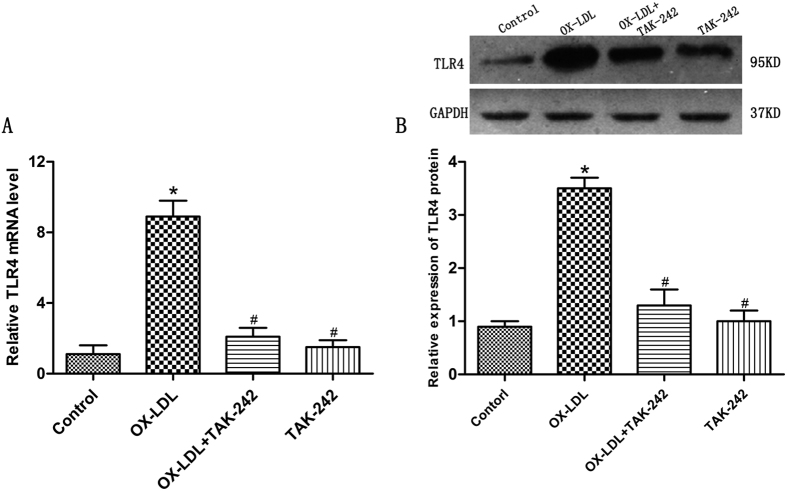
Examination of TLR4 expression level in each group. (**A**) Changes of TLR4 mRNA in each group by fluorescent quantitative PCR; (**B**) Changes of TLR4 protein in each group as determined by western blot. Data are expressed as mean ± standard deviation(n = 4). *P < 0.05 versus the control group; ^#^*P* < 0.05 versus ox-LDL-treated group. TLR4, toll-like receptor 4; GAPDH, glyceraldehyde 3-phosphate dehydrogenase.

**Figure 4:**
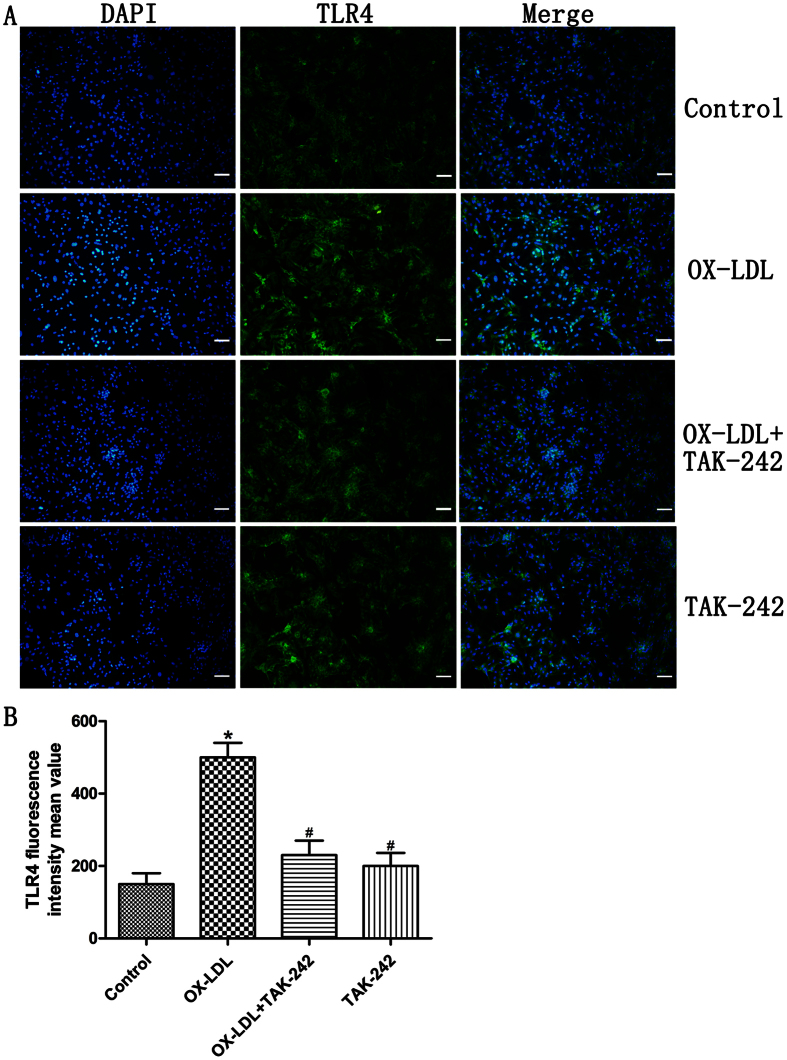
Immunofluorescence of TLR4 in each group. (**A**) Representive immunofluorescence images of cardiomyocytes stained with TLR4 (green) and nucleus (blue) under x100 magnification. DAPI was used for nuclear counterstaining. (**B**) Determination of TLR4 fluorescence intensity mean value. Each group contains six samples. Data are mean ± standard deviation. *P < 0.05 versus the control group; ^#^*P* < 0.05 versus ox-LDL-treated group. Scale bar = 100 μm. TLR4, toll-like receptor 4; DAPI, 4′,6-diamidino-2-phenylindole.

To observe whether ox-LDL increased the expression of TLR4 in cultured cardiomyocytes, cells were incubated with 50 mg/L ox-LDL for 24 h. RT-PCR and western blot analysis revealed that mRNA and protein levels of TLR4 were increased after being treated with ox-LDL (Fig. 3). Meanwhile, the protein and mRNA levels of TLR4 were down-regulated by pretreatment with TAK-242. (Fig. 3). The immunofluorescence results were consistent with RT-PCR and western blot analysis (Fig. 4).

### Effect of ox-LDL on NF-κB, Bcl-2/Bax ratio and caspase activation in cardiomyocytes

**Figure 5:**
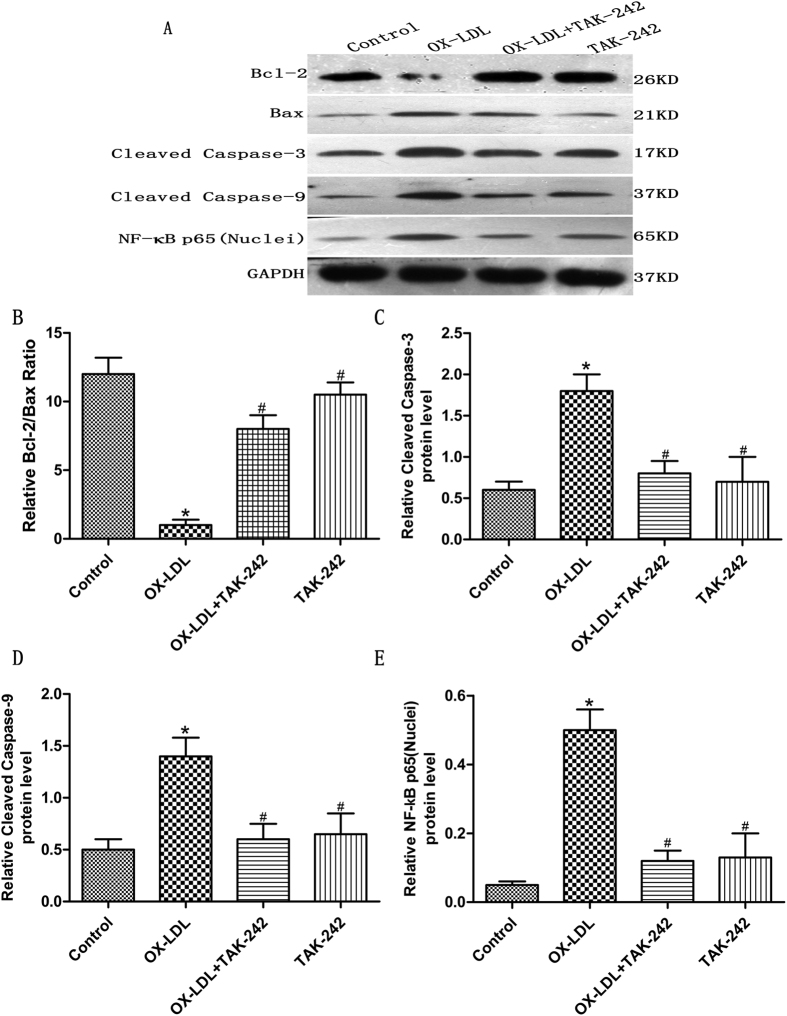
Protein expression of Bcl-2, Bax, Cleaved caspase-3, Cleaved caspase-9 and NF-κB p65 (nuclei) in ox-LDL treated cardiomyocytes by western blot analysis. (**A**) Representative Western blots. (**B–E**)Protein expression relative to GAPDH of (**B**) Bcl-2/Bax ratio, (**C**) Cleaved caspase-3, (**D**) Cleaved caspase-9, and (**E**) NF-κB p65 (nuclei); mean ± standard deviation (n = 4 for each group). Pretreatment with TAK-242 reversed the expression of these proteins. *P < 0.05 versus the control group; ^#^*P* < 0.05 versus ox-LDL-treated group. GAPDH, glyceraldehyde 3-phosphate dehydrogenase.

NF-κB activation is an early step downstream of TLR4 signaling pathway, accordingly, the effect of ox-LDL on NF-κB activation was investigated by western blot analysis. To explore whether ox-LDL-induced apoptosis was associated with Bcl-2 and Bax in cultured cardiomyocytes, we also detected the alterations in the expression of Bcl-2 and Bax proteins by western blot analysis. Caspase-3 and caspase-9 are important mediators of cell apoptosis, therefore, we determined the activity of caspase-3 and caspase-9 by Western blot. Our results showed that ox-LDL markedly increased protein levels of NF-κB p65(nuclei), cleaved caspase-3 and -9. Furthermore, the stimulation of cells with ox-LDL produced a significant decrease of the Bcl-2/Bax ratio compared to control group. Pretreatment of cells with TAK-242 before the cells were exposed to ox-LDL significantly inhibited the activation of NF-κB, caspase-3 and -9, and the decrease in the Bcl-2/Bax ratio. (Fig. 5)

## Discussion

It has been reported that TLR4 signaling plays an important role in the cell response to ox-LDL stimulation^11^,^12^,^13^,^14^,^15^,^16^.The plasma level of ox-LDL is an important prognostic marker in atherosclerosis and chronic congestive heart failure patients^5^,^17^. Ox-LDL can induce apoptosis in a variety of cell types and ox-LDL-mediated apoptosis may participate in the progression of atherosclerosis, and the final acute cardiovascular events^17^,^18^,^19^. Ox-LDL was found to induce severe cell damage and irregular electrical activity in adult ventricular myocytes^6^. However, the role of TLR4 in ox-LDL-induced apoptosis in cardiomyocytes was not clearly defined yet. The present study revealed that ox-LDL induces apoptosis of cultured neonatal rat cardiomyocytes by up-regulating the expression of TLR4. Additionally, our results showed that the activation of TLR4/NF-κB signaling pathway was a potential mechanism for ox-LDL-induced apoptosis in cardiomyocytes. The TLR4 inhibitor, TAK-242, significantly attenuated the ox-LDL-induced apoptosis of primary myocardial cells.

It has been reported that plasma levels of ox-LDL correlate with the development of atherosclerosis and its complications^20^. Toll-like receptors play a pivotal role in host immune defense against invading pathogens and endogenous danger signals such as ox-LDL in various tissues including the heart^21^,^22^. TLR4 has been demonstrated to mediate myocardial ischemia reperfusion injury, maladaptive left ventricular remodeling and increased infarct size after myocardial infarction^23^,^24^,^25^. Some research findings suggest that ox-LDL activates TLR4 signaling pathway, which is considered to be a promising therapeutic target for the treatment of atherosclerotic cardiovascular diseases^11^,^12^,^13^,^14^,^15^,^16^,^26^. In the present study, we revealed that ox-LDL up-regulates mRNA and protein levels of TLR4.

Nowicki *et al.* have found that ox-LDL-induced apoptosis in dorsal root ganglion cell cultures depends on TLR4^9^. In this study, ox-LDL-mediated apoptosis is reduced by pretreatment with TAK-242, an exogenous synthetic antagonist for TLR4, suggesting that the TLR4 pathway plays a critical role in ox-LDL-induced apoptosis. Apoptosis in the present study was documented by flow cytometry. The results were consistent with characteristic changes in markers of apoptosis including Bcl-2, Bax, Cleaved caspase-3 and 9 proteins.

NF-κB plays a crucial role in cardiac pathological processes via regulating expression of many genes that are involved in apoptosis of cardiomyocytes^27^.NF-κB activation is an early step downstream of TLR4 signaling pathway and is pivotal in the transcription and translation of caspases expression. Research data *in vitro* and vivo suggested the potential importance of TLR4/NF-κB pathway in myocardial ischemia-reperfusion injury^12^,^27^. Therefore, inhibition of TLR4-mediated NF-κB activation could be an important approach for attenuation of cardiac dysfunction.

Caspases play a vital role in the regulation and execution of apoptosis. We determined the activity of caspase-3 and caspase-9 in the present study to examine downstream apoptotic signaling during ox-LDL-induced apoptosis. Caspase-3 catalyzes a terminal step in apoptosis, and its activation may serve as a marker of apoptosis in cardiomyocyte^28^. It is well known that Bcl-2 family, including anti-apoptotic Bcl-2 and pro-apoptotic Bax proteins has been shown to play a major role in regulating the possibility of cells to survive or undergo apoptosis after a certain stimulus or injury^29^. The results of the present study showed that ox-LDL significantly increased the activation of caspase-3and 9, and expression of Bax, and significantly reduced the expression of Bcl-2.

Atherosclerotic diseases exhibit serious complications on a number of different systems. The present study predominantly focused on ox-LDL induced myocardial cells injury and investigated the possible underlying mechanisms. We examined the direct effects of ox-LDL on primary myocardial cells of neonatal rat. Ox-LDL induced myocardial cells injury was shown to be associated with TLR4/NF-κB signaling pathway activation-induced cell apoptosis. These findings may provide new therapeutic options for myocardial injury in atherosclerotic cardiovascular diseases.

For better interpretation of the results, some limitations in this study should be acknowledged. First, the results were derived from neonatal rat cardiomyocytes; therefore, as with all *in vitro* studies, the current results may not be directly comparable with those obtained *in vivo* setting. Second, differences may exist between neonatal and adult cardiomyocytes. Third, the biological properties of isolated cardiomyocytes in culture and those in organized hearts *in vivo* may differ. Considering of this, future studies are therefore required to explore *in vivo* setting and in adult cardiomyocytes.

## Materials and Methods

### Cell culture and treatment groups

All animal procedures were conducted in accordance with the Guide for the Care and Use of Laboratory Animals (NIH Publication No. 85-23, revised 1996) and were approved by the Animal Care and Use Committee of Guangxi Medical University. Primary cardiomyocytes were isolated enzymatically with collagenase II (Sigma, USA) from 1–3 days old Sprague-Dawley rat ventricles (Medical Experimental Animal Center of Guangxi Medical University, China) according to a previous protocol^30^. Briefly, the ventricles were minced and digested with 0.04% collagenase II, and then the supernatant-containing suspended cells were preplated for 1.5 h to remove non-myocytes. The isolated cardiomyocytes were seeded onto cell culture plates at approximately 5 × 10^4^ cells/cm^2^ and cultured in medium containing DMEM/F-12 with HEPES(Hyclone, Beijing, China), 10%FBS (Gibco, Australia) and 1% penicillin-streptomycin (Solarbio, Beijing, China) at 37 °C with 5% CO_2_. Three days after being seeded, the cardiomyocytes were incubated with different concentrations of ox-LDL (0, 10, 50, 100 mg/L) for 24 h or with 50 mg/L ox-LDL for 0, 6, 12 and 24 h respectively at identical environment. Ox-LDL was obtained from Yiyuan Biotechnology (Guangzhou, China). Gel qualitative method confirmed the ox-LDL was free from endotoxins.

The cardiomyocytes were grouped randomly as follows: Group A: Control group, Group B: Ox-LDL group, Group C: Ox-LDL + TAK-242 (1 μM) group and Group D: TAK-242 group. TAK-242 was purchased from MedChem Express (Princeton, USA).

### Flow cytometry assay

Flow cytometry was conducted to detect cell apoptosis using an Annexin V-FITC and propidium iodide (PI) double staining kit (Shanghai BestBio Biotech. Co. Ltd., Shanghai, China) according to the manufacturer’s instructions. Briefly, cells were collected after treatment as indicated, washed with ice-cold PBS, resuspended in 400 μl of binding buffer, then incubated with 5 μl of Annexin V-FITC and 5 μl of PI at room temperature in the dark for 15 min. Samples were analyzed by a flow cytometer within 1 h (BD Biosciences, San Jose, CA, USA). All the cells stained positively for Annexin V-FITC were considered apoptotic cells.

### Immunofluorescence

After being treated as indicated, the cardiomyocytes cultured on glass coverslips in 24-well plates were washed with PBS for three times, fixed with 4% paraformaldehyde in PBS for 15 min at room temperature, permeabilized with 0.1% Triton X-100 for 10 min, and then blocked with 5% BSA for 1 h.Then, cardiomyocytes were incubated overnight at 4 °C with monoclonal mouse anti-rat TLR4 antibody (1:100; Abcam, Cambridge, USA). After being washed with PBS for three times, cardiomyocytes were incubated with FITC-conjugated goat anti-mouse polyclonal IgG (1:400; Abcam,Cambridge, USA) at room temperature in the dark for 2 h. For nuclear counterstaining, cardiomyocytes were incubated with 4′,6-diamidino-2-phenylidone (DAPI; Sigma, USA) for 5 min.

At last, the immunofluorescence images were obtained by inverted fluorescence microscope (Olympus, Tokyo, Japan). The TLR4 fluorescence intensity mean values were determined using the Image-Pro Plus software 6.0 (Media Cybernetics).

### Quantitative RT-PCR analysis

Total RNA was extracted from cardiomyocytes using the TRIzol reagent (Gibco, USA) according to the protocols supplied by the manufacturers. The concentration of RNA was quantified by a NanoDrop (Thermo Fisher Scientific Inc., USA) and subjected to reverse transcription using a cDNA reverse transcription kit (Promega, USA) according to the manufacturer’s instructions. Then, the obtained cDNA was subjected to RT-PCR for TLR4 mRNA using a SYBR Green PCR kit (Promega, USA). The conditions for all RT-PCR reactions were performed on the ABI PRISM 7500 system (Applied BioSystems, USA). The sequences of the primers were designed as follows: TLR4 forward: 5′-AAGTTATTGTGGTGGTGTCTAG-3′ and reverse: 5′-GAGGTAGGTGTTTCTGCTAAG-3′; GAPDH forward: 5′-TGCACCACCAACTGCTTAG-3′ and reverse: 5′-GATGCAGGGATGATGTTC-3′. The relative quantification of TLR4 mRNA expression was calculated using the 2^−∆∆Ct^ method and was normalized to GAPDH.

### Western blot analysis

The protein samples were extracted from cardiomyocytes with Protein Extracion Kit (Solarbio, Beijing, China). The protein concentrations were measured by a nanodrop instrument (Thermo Fisher Scientific Inc., USA). Each sample containing equal amounts (20 μg) of protein was loaded on 10% SDS-PAGE and transferred to a PVDF membrane (Millipore). After blocking with 5% non-fat milk in TBS for 1 h at room temperature, membranes were incubated with the following primary antibodies in dilution buffer overnight at 4 °C: anti-TLR4, anti-NF-κB (p65), anti-Bcl-2, anti-Bax, anti-cleaved caspase-3, anti-cleaved caspase-9 and anti-GAPDH (all from Abcam, Cambridge,USA). GAPDH was used as an internal control. After washing three times in TBST, the membranes were incubated with goat anti-mouse horseradish peroxidase (HRP)-conjugated secondary antibodies (KeyGEN BioTECH, Nanjing, China) in dilution buffer for 2 h at room temperature. Then, antigen-antibody complex was visualized using the enhanced chemiluminescence (ECL) method. Immunoblots were exposed to X-ray film and images were taken using an imaging system (BioRad, USA). At last, the results were analyzed using the Quantity One software (Bio-Rad).

### Statistical analysis

All data are presented as mean ± standard deviation (SD). Differences between multiple groups were analyzed by one-way ANOVA followed by Student-Neuman-Keuls or Dunnett test, using Prism software (GraphPad Prism version 5.0). All results presented are representative of multiple experiments. All experiments were performed independently in quadruplicate. Values of P < 0.05 were considered as statistically significant.

## Additional Information

**How to cite this article**: Wang, X. *et al.* Oxidized Low-Density Lipoprotein Induces Apoptosis in Cultured Neonatal Rat Cardiomyocytes by Modulating the TLR4/NF-κB Pathway. *Sci. Rep.* **6**, 27866; doi: 10.1038/srep27866 (2016).
